# Use of Tocilizumab in COVID-19: A Systematic Review and Meta-Analysis of Current Evidence

**DOI:** 10.7759/cureus.10869

**Published:** 2020-10-09

**Authors:** Sohny Kotak, Mahima Khatri, Mehreen Malik, Maria Malik, Warda Hassan, Arooba Amjad, Farheen Malik, Hani Hassan, Jawad Ahmed, Marium Zafar

**Affiliations:** 1 Internal Medicine, Dow University of Health Sciences, Karachi, PAK; 2 Anesthesiology, Aga Khan University, Karachi, PAK; 3 Anesthesiology, Indus Hospital, Lahore, PAK; 4 Internal Medicine, Karachi Medical and Dental College, Karachi, PAK; 5 Internal Medicine, Dow Medical College, Karachi, PAK

**Keywords:** covid-19, sars-cov-2, interleukin-6, tocilizumab, systematic review, meta-analysis, coronavirus disease, mortality, safety, efficacy

## Abstract

Background and objectives

A flare-up in coronavirus disease 2019 (COVID-19) cases threatens the health of people, and though there is no proven pharmacological treatment, many analytical studies have suggested that interleukin-6 (IL-6) levels are elevated in cases of severe COVID-19 and that the anti-IL-6 biologic agent tocilizumab may be beneficial. This is a critical review of studies aiming to assess the safety and efficacy of tocilizumab as compared to the standard regimen in patients with COVID-19.

Materials and methods

Online databases (PubMed and Cochrane) were searched until June 29, 2020, for original articles investigating the immunological response in COVID-19 and its treatment with tocilizumab. Data on multiple baseline characteristics and pre-specified endpoints were extracted and pooled using a random effect model. We used Review Manager version 5.3 (The Nordic Cochrane Centre, The Cochrane Collaboration, 2014, Denmark) and Stata 11.0 (Stata Corporation LP, College Station, TX) for all analyses. Risk ratios (RR) and the weighted mean difference (WMD) with the corresponding 95% confidence interval (CI) were calculated.

Results

From a total of 1,246 identified articles, 13 studies were included after duplicate removal and narrowing based on title and abstract. Of the 13 included studies, seven case-control studies were shortlisted for meta-analysis (quantitative) and six-single arm studies were used in the discussion (qualitative). These studies had 766 patients (351 in the tocilizumab arm and 414 in the control arm). Their pooled analysis demonstrated that mortality was significantly lower in the tocilizumab group (RR=0.56 [0.34, 0.92]; p=0.02; I^2^=76%), as was the need for artificial invasive ventilation (RR=0.34 [0.12, 0.99]; p=0.05; I^2^=0%) as compared to the control group. No significant differences were observed between tocilizumab and control group in intensive care unit admissions (RR=0.73 [0.15, 3.59]; p=0.70; I^2^=60%) and risks of post-drug infection (RR=1.29 [0.41, 4.04]; p=0.66; I^2^=88%). In terms of efficacy outcome, improved oxygen saturation (RR=1.13 [1.04, 1.65]; p=0.02; I^2^=0%) was reported to be markedly significant in tocilizumab patients when compared with the standard care group.

Conclusions

Our results based on pooled studies show tocilizumab to be safe and efficacious in reducing mortality among critically ill COVID-19 patients. However, due to the limited number of observational studies, the positive findings should be viewed cautiously and warrant further investigation.

## Introduction

An exponential increase in the number of patients infected by severe acute respiratory syndrome-related coronavirus (SARS-CoV-2), as well as the rapidly changing disease profile of coronavirus disease 2019 (COVID-19), has been a life-threatening and public health emergency [1]. Despite several disease complications, including gastrointestinal and neurological complications, the leading cause of mortality remains pulmonary failure [2]. Thus, in the quest for developing urgent and effective treatment options for COVID-19 pneumonia, it is crucial to explore treatment options that can slow its progression, reduce the rapid rate of hospitalizations and intensive-care unit (ICU) admissions, reducing the burden on public health systems; and mortality. The current treatment is either supportive or investigational based on interim data from studies that are underway.

Although its pathogenesis is still vague, laboratory findings of patients suffering from severe disease show a cytokine storm, involving a considerable release of pro-inflammatory cytokines, among which Interleukin-6 (IL-6) plays a cardinal role [3]. Tocilizumab, a humanized monoclonal antibody, is one of its kind against the IL-6 receptors and may turn down this exaggerated inflammatory response in critically ill patients [4]. Thus, the National Health Commission of China, in its latest 7th version, has advocated the use of tocilizumab in critically ill patients with elevated IL-6 levels [5].

Studies carried out to evaluate the adequacy of tocilizumab so far have yielded different results. Some observational studies showed it to be beneficial, which led to its use in some countries and prompted further research; however, a recent randomized controlled trial (RCT), COVACTA (A Study to Evaluate the Safety and Efficacy of Tocilizumab in Patients With Severe COVID-19 Pneumonia; NCT04320615), suggested no evidence of its benefit in COVID-19 [6]. Hence, the available literature was critically reviewed to find possible reasons for these differences. Additionally, data from different observational studies were pooled and analyzed to report more robust conclusions on the outcomes of tocilizumab in critically ill patients infected with COVID-19 contrasted with the accepted regimen being followed worldwide.

## Materials and methods

This meta-analysis was conducted according to the guidelines set by Preferred Reporting Items for Systemic Review and Meta-Analysis (PRISMA) and Cochrane guidelines [7-8].

Search strategy

A systematic literature search was conducted up till June 29, 2020, on the PubMed and Cochrane CENTRAL databases with the following subject keywords and their MeSH terms: ((COVID-19 OR Coronavirus OR SARS-coV-2) AND (Tocilizumab OR IL-6 antagonists OR IL-6 Inhibitors).

Google Scholar and Clinicaltrials.gov were searched for any studies that had not yet been published but had reported their results online. There was no language barrier, as all the studies retrieved in the search were in the English language. Two reviewers independently screened the search results. A third reviewer was consulted in case of discrepancies. Duplicates were removed and studies were initially shortlisted based on title and abstract, after which the full text was assessed for eligibility. References of the selected studies were also reviewed thoroughly to prevent any risk of selection bias.

Inclusion and exclusion criteria

Observational studies, including adults ≥18 years with polymerase chain reaction (PCR)-confirmed SARS-CoV-2 infection in which the efficacy of tocilizumab added to standard therapy and standard therapy alone, were compared. Additionally, studies that had published full-text in the English language were considered. Only articles were excluded if they were reviews, editorials, or case reports. All identified studies were imported in EndNote Reference Library version X4 (Clarivate Analytics, Thomson Reuters Corporation, Philadelphia, Pennsylvania) for the removal of duplicates.

Data extraction

Data extraction of the relevant studies included the first author, year of publication, type of study, study follow-up time, total number of COVID-19 positive patients, and patients who received tocilizumab. From the obtained relevant studies, the baseline characteristics (such as age, gender, and standard therapy) and comorbidities (such as heart disease, hypertension, diabetes mellitus, etc.) of patients in the two groups were also extracted. We accepted the study investigator’s definition for all safety and efficacy outcomes. The outcomes extracted are mentioned below.

Safety outcomes

Safety outcomes included mortality, ICU admission, risk of infection (bacteremia, fungemia, and candidiasis), need for mechanical ventilation, and serious adverse events.

Efficacy outcome

Improved oxygen saturation was considered an efficacy outcome.

Assessment of risk of bias

Quality assessment of all the observational studies was done using the Newcastle-Ottawa reference scale [9].

Statistical analysis

Statistical analysis was done only for comparative studies using Review Manager version 5.3 (The Nordic Cochrane Centre, The Cochrane Collaboration, 2014, Denmark) and Stata 11.0 (Stata Corporation LP, College Station, TX). Relative risks (RR) with 95% confidence intervals (CIs) were calculated using raw study data and were pooled using a random-effects model for dichotomous data. Due to the lack of adjusted outcome measures, baseline differences were assessed between the tocilizumab and control group patients in this study to identify potential confounding factors. Funnel plots for all outcomes were visualized and Begg's test was performed to assess publication bias. Potential causes of heterogeneity were explored by carrying out a subgroup analysis according to the use of corticosteroids analyses. The Higgins I^2^ statistic was used to evaluate heterogeneity, and the value of 25%-50% as mild, 50%-75% as moderate, and >75% was considered significant heterogeneity. A p-value of ≤0.05 was considered significant.

## Results

**Figure 1:**
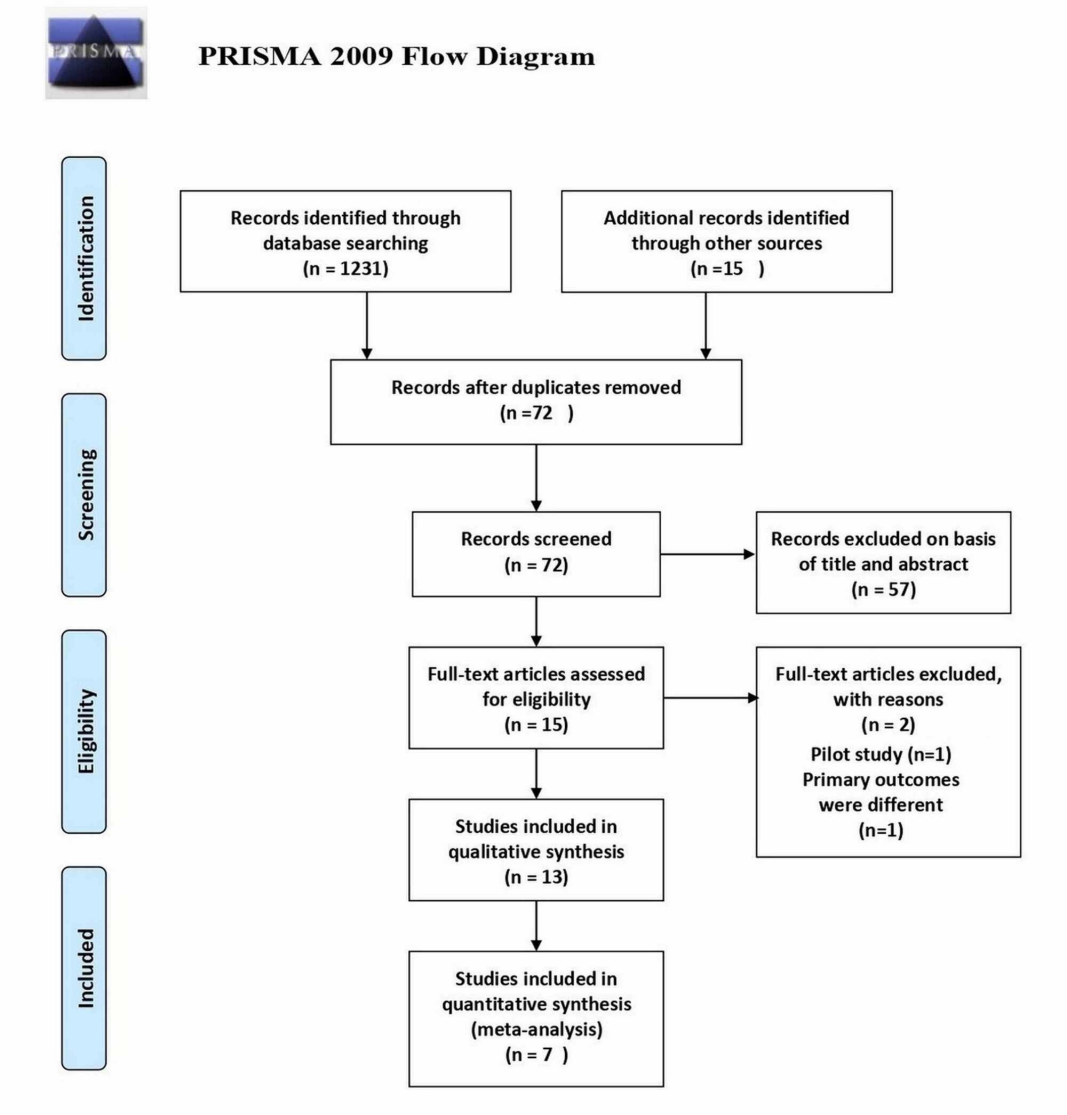
PRISMA flow chart for study selection PRISMA: Preferred Reporting Items for Systemic Review and Meta-Analysis

 A total of 1,246 articles were identified initially from the literature search, 1,231 from database searches and 15 from other sources such as references of relevant studies. Finally, 13 retrospective observational studies were found, amongst which seven were comparative [10-16] and six were single-arm [17-22]. In this meta-analysis, only seven comparative studies were included. A detailed search is illustrated in the PRISMA flow-chart (Figure 1).

Quality assessment and publication bias

**Figure 2:**
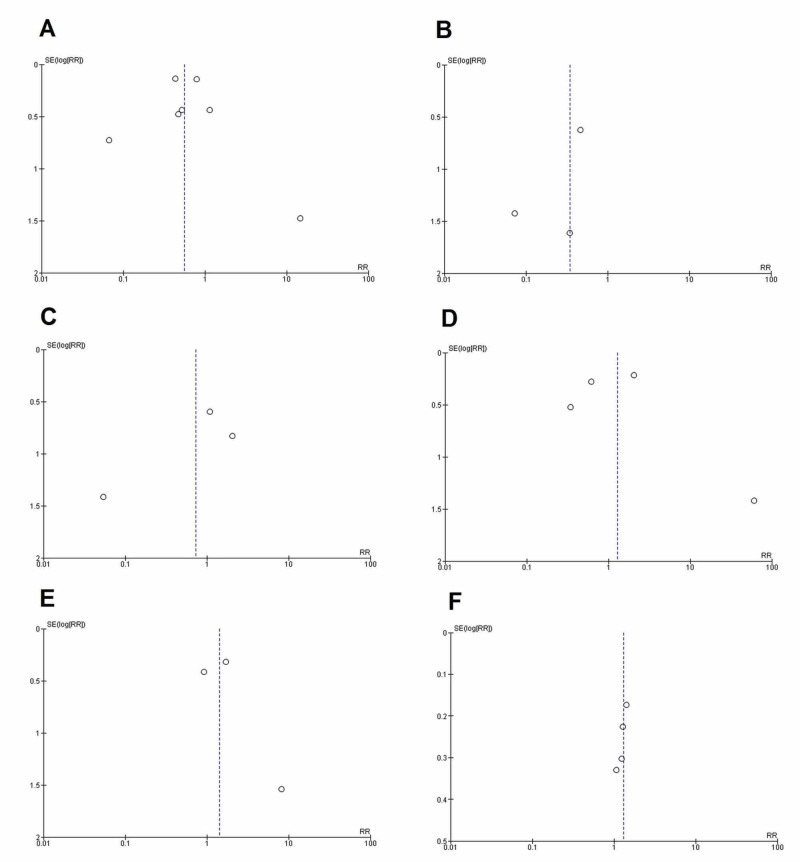
Funnel plots for publication bias (A) Mortality; (B) Post-therapy mechanical ventilation; (C) ICU admission; (D) Frequency of post-drug infection; (E) Serious adverse events; and (F) Improved oxygenation. SE, standard error; RR, risk ratio; ICU, intensive care unit

The quality assessment of studies using the New Castle Ottawa scale depicted a significantly low risk of bias in all the included case-control studies (Table 1). The funnel plots showed no publication bias (Figure 2), which was confirmed by Begg’s test with the exception of improved oxygen saturation (efficacy outcome). The details of Begg’s test for all outcomes is given in Table 2.

**Table 1:** Quality assessment of the included observational studies

Author	Cohort representation	Selection of non-exposed cohort	Ascertainment of exposure	Outcome not present at baseline	Comparability of cohorts for important factors	Comparability of cohorts for other variables	Assessment of outcome	Follow-up long enough for an outcome to occur	Adequacy of follow- up	Overall risk of bias	
Corrado Campochiaro et al. [16]	1	1	1	1	1	…	1	…	1	Low	
T. Klopfenstein et al. [10]	1	1	1	1	1	…	1	…	1	Low	
Marta Colaneri et al. [15]	1	1	1	1	1	1	1	…	1	Low	
Geurys R. Rojas-Marte et al. [12]	1	1	1	1	1	1	1	…	1	Low	
Ruggero Capra et al. [11]	1	1	1	1	1	…	1	…	1	Low	
Luca Quartuccio et al. [13]	1	1	1	1	1	…	1	…	1	Low	
Emily C Somers et al. [14]	1	1	1	1	1	1	1	…	1	Low	

**Table 2:** Results of Begg’s test of publication bias for all outcomes

Category	Outcomes	Begg’s p-value
Safety outcomes	Mortality	0.881
	Post-therapy mechanical ventilation	0.317
	ICU admission	0.317
	Post-drug infection	1.000
	Serious adverse events	0.602
Efficacy outcomes	Improved oxygen saturation	0.042

Baseline characteristics

The seven studies included 766 patients (351 in the tocilizumab arm and 414 in the control arm). The baseline characteristics and comorbidities of the comparative studies are given in Table 3 and Table 4, respectively.

**Table 3:** Baseline characteristics of the case-control studies included in the meta-analysis bid, twice a day; IU, international units

Study	Type of study	Follow-up period (days)	Total No. of Pts	Patients receiving tocilizumab (n)	Male (n)		Age (years)		Standard therapy
					Tocilizumab group	Control group	Tocilizumab group	Control group	
Rojas-Marte et al. [12]	Retrospective, case-control, single-center study	48	193	96	74	63	58.8 ± 13.6	62.0 ± 14	Hydroxychloroquine, azithromycin, corticosteroids, remdesivir, and anticoagulation
Marta Colaneri et al. [15]	Retrospective, single-center	7	112	21	19	63	62.33 ± 18.68	63.74 ± 16.32	Combination of hydroxychloroquine (200 mg bid), azithromycin (500 mg once), a prophylactic dose of low weight heparin, and methylprednisolone (a tapered dose of 1 mg/kg up to a maximum of 80 mg) for 10 days.
Klopfenstein et al. [10]	Retrospective, case-control, single-center study	13	45	20	NR	NR -	76.8 ± 11	70.7 ± 15	Hydroxychloroquine or lopinavir-ritonavir therapy and antibiotics, and less commonly corticosteroids
Corrado Campochiaro et al. [16]	Retrospective, case-control, single-center study	28	65	32	29	27	64 (53 – 75)	60 (55 – 75.5)	Hydroxychloroquine 400 mg, daily, lopinavir/ritonavir 400/100 mg twice daily, ceftriaxone 2 gr for 6 days, azithromycin 500 mg daily until a negative report of urine. enoxaparin 4000 IU subcutaneously once a day
Ruggero Capra et al. [11]	Retrospective	21	85	62	45	19	63 (54-73)	70 (55-80)	Hydroxychloroquine 400 mg daily and lopinavir 800 mg daily plus ritonavir 200 mg daily
Luca Quartuccio et al. [13]	Single-center retrospective study	38	111	42	33	44	62.4 ± 11.8	56.2 ± 14.2	Lopinavir/ritonavir, darunavir/cobicistat, remdesivir, hydroxychloroquine, chloroquine, and methylprednisolone
Emily C Somers et al. [14]	Single-center retrospective study	47	154	78	53	49	55 ± 14.9	60 ± 14.5	Hydroxychloroquine, remdesivir, NSAIDs, acetaminophen, ace inhibitors or angiotensin receptor blockers, vasopressors, anticoagulants, oral prednisone, and methylprednisolone

**Table 4:** Comorbidities of the case-control studies included in the meta-analysis TCZ, tocilizumab; COPD, chronic obstructive pulmonary disease; NR, not reported

Study	Heart disease n (%)		Hypertension n (%)		COPD n (%)		Diabetes mellitus n (%)	
	TCZ group	Control group	TCZ group	Control group	TCZ group	Control group	TCZ group	Control group
Rojas-Marte et al. [12]	11 (11.5)	18 (18.5)	53 (55.2)	51 (52.6)	8 (8.3)	3 (3.1)	29 (30.2)	38 (39.2)
Marta Colaneri et al. [15]	2(9.5)	7(7.7)	8(38)	20 (21.9)	0	4(19)	2 (9.5)	8 (8.8)
Klopfenstein et al. [10]	14 (70)	17 (68)	11 (55)	11 (44)	4 (20)	1 (4)	5 (25)	8 (32)
Corrado Campochiaro et al. [16]	4 (12)	6 (18)	12 (37)	16 (48)	1 (3)	2 (6)	4 (12)	6 (18)
Ruggero Capra et al. [11]	8 (14)	6 (26)	28 (46)	11 (488)	88	88	88 (14)	5 (22)
Luca Quartuccio et al. [13]	NR	NR	20 (47.6)	21 (30.4)	NR	NR	NR	NR
Emily C Somers et al. [14]	16 (21)	20 (26)	50 (64)	52 (68)	8 (10)	21 (28)	10 (13)	15 (20)

Assessment of baseline differences

**Figure 3:**
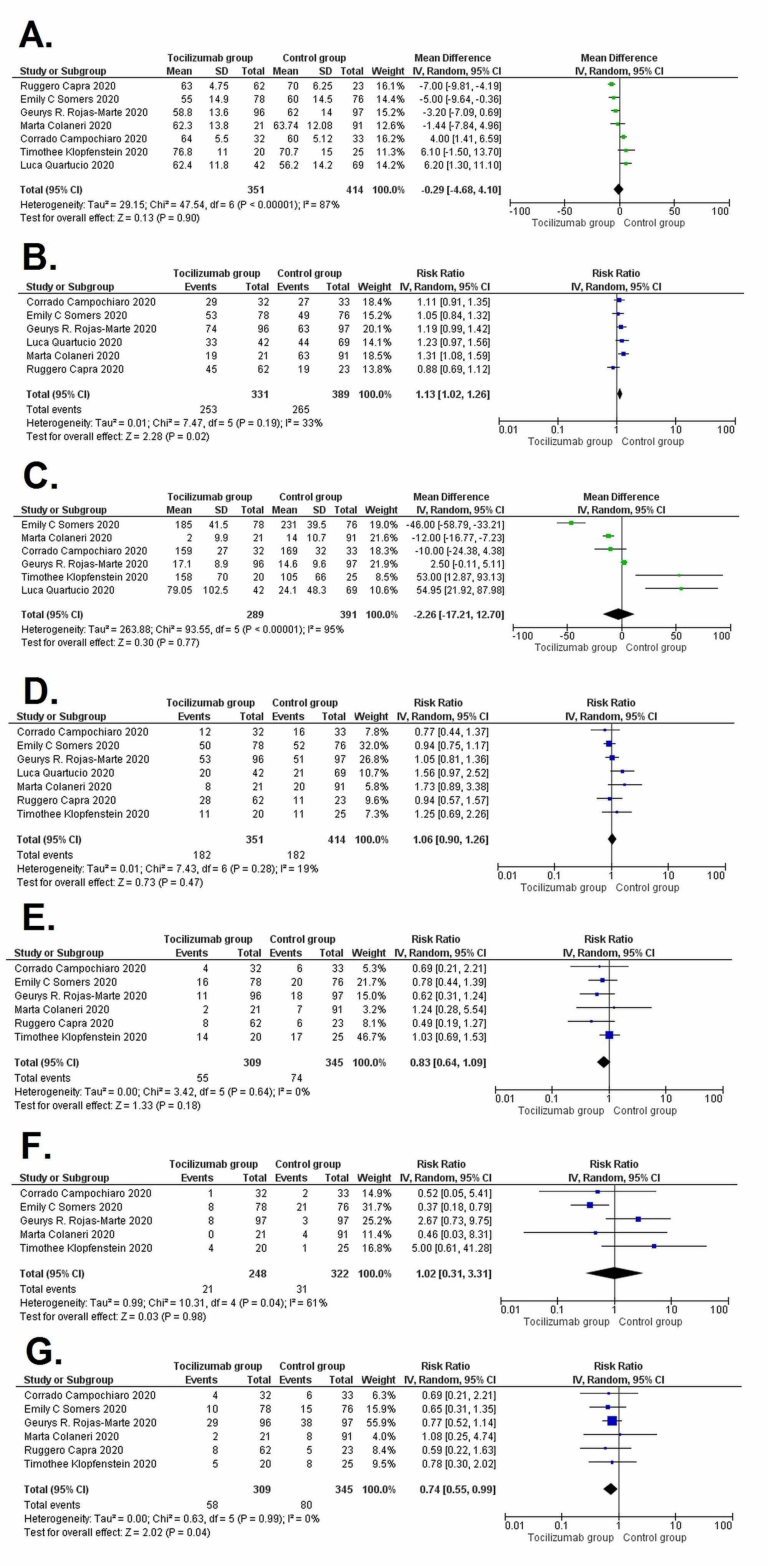
Forest plots showing pooled baseline demographics comparing tocilizumab group and control group patients (A) Age; (B) Males; (C) C-reactive protein levels; (D) Hypertension; (E) Heart disease; (F) Chronic obstructive pulmonary disease; and (G) Diabetes mellitus

When baseline characteristics were pooled, we found significantly more male patients (RR= 1.13 [1.02, 1.26]; p=0.02) and less diabetic patients (RR= 0.74 [0.55, 0.99]; p=0.04) in the tocilizumab group. All the remaining characteristics were insignificant between both the intervention groups. Differences in key baseline characteristics between the tocilizumab group and the control group are represented in Table 5. The forest plots for the baseline assessment difference are given in Figure 3.

**Table 5:** Pooled baseline demographics comparing tocilizumab group and control group patients CRP, C-reactive protein; COPD, chronic obstructive pulmonary disease; RR, relative risks; WMD, weighted mean difference; CI, confidence interval

Baseline characteristics	Tocilizumab vs. control patients [95% CI]	p-value
Age	WMD= -0.29 [-4.48, 4.10]	0.90
Males	RR= 1.13 [1.02, 1.26]	0.02
CRP levels (mg/dL)	WMD = -2.26 [-17.21, 12.70]	0.77
Hypertension	RR= 1.06 [0.90, 1.26]	0.47
Heart disease	RR= 0.83 [0.64, 1.09]	0.18
COPD	RR= 1.02 [0.31, 3.31]	0.98
Diabetes mellitus	RR= 0.74 [0.55, 0.99]	0.04

Safety outcomes

**Figure 4:**
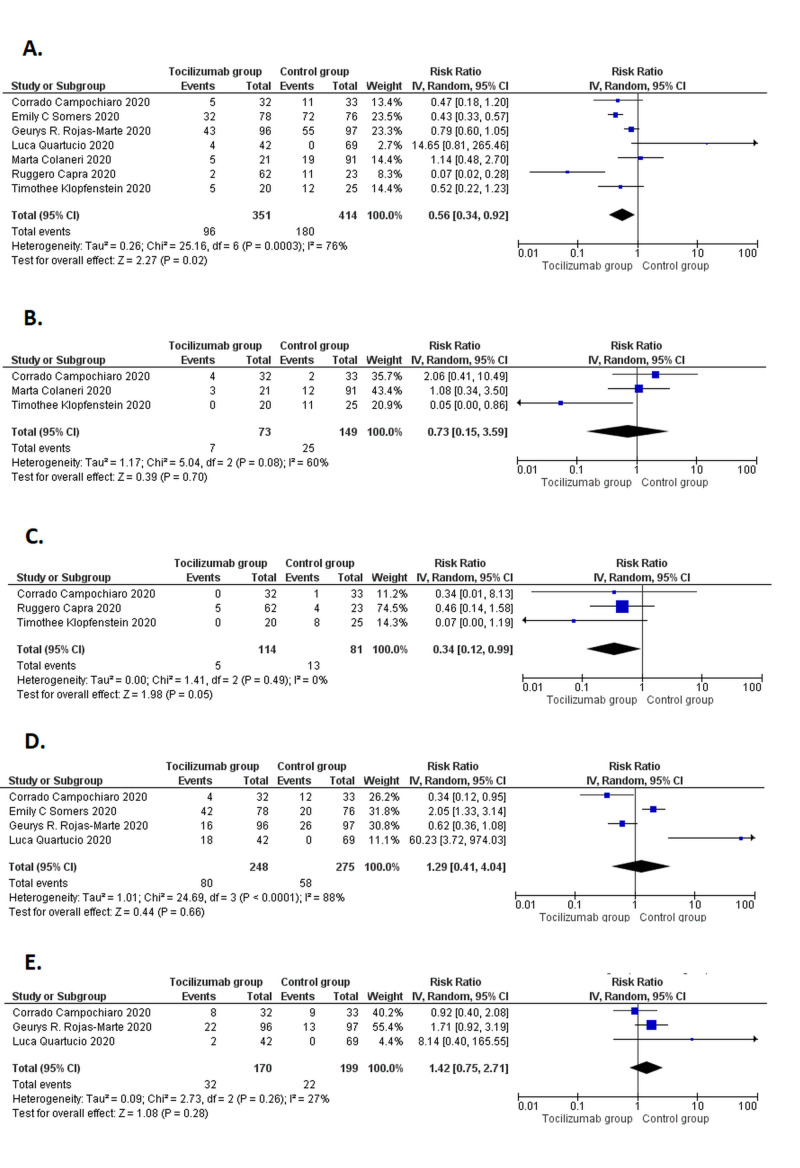
Forest plots for safety outcomes (A) Mortality; (B) Intensive care unit admission; (C) Post-therapy mechanical ventilation; (D) Post-drug infection; and (E) Serious adverse events CI, confidence interval; IV, inverse variance; M-H, Mantel-Haenszel The studies used in the analysis include [10-16].

The pooled analysis of the patients receiving tocilizumab showed a significantly lower risk of mortality (RR=0.56 [0.34, 0.92]; p=0.02; I^2^=76%) as compared to the control group, and no publication bias was found for the mortality outcome (p=0.20). Also, there was a significant decrease in patients needing post-therapy mechanical ventilation (RR=0.34 [0.12, 0.99], p=0.05, I^2^=0%) in the tocilizumab group versus the control group. Yet, no significant differences were observed between the two groups in terms of ICU admissions (RR= 0.73 [0.15, 3.59]; p=0.70, I^2^=60%), risk of post-drug infection (RR= 1.29 [0.41, 4.04]; p=0.66; I^2^=88%) and serious adverse events (RR= 1.42[0.75, 2.71]; p=0.28; I^2^=27%). The forest plots for all the above-mentioned outcomes are displayed in Figure 4.

Efficacy outcome

**Figure 5:**
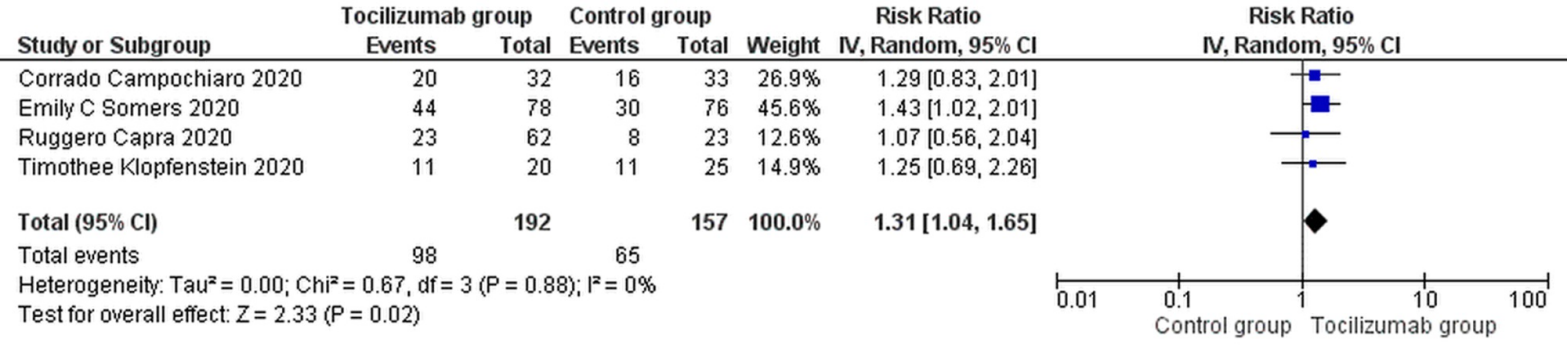
Forest plots for efficacy outcome (improved oxygenation) CI, confidence interval; IV, inverse variance; M-H, Mantel-Haenszel Studies used in the analysis include [10-11,14,16]

However, in terms of efficacy outcome, improved oxygen saturation (RR=1.13 [1.04, 1.65]; p=0.02; I^2^=0%) was reported to be markedly significant in tocilizumab patients when compared with the standard care group as shown in Figure 5.

Subgroup analysis by corticosteroid use

**Figure 6:**
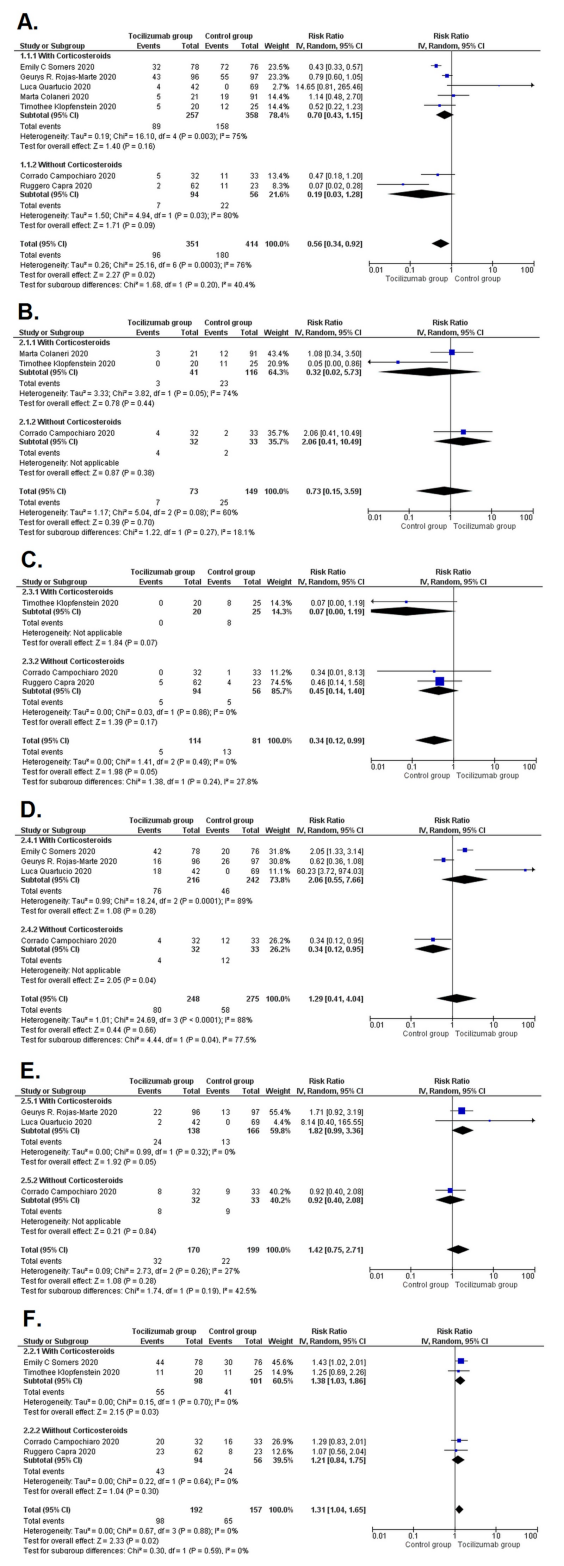
Forest plots showing subgroup analysis by corticosteroid use for all outcomes (A) Mortality; (B) Intensive care unit admission; (C) Post-therapy mechanical ventilation; (D) Post-drug infection; (E) Serious adverse events; and (F) Improved oxygen saturation. CI, confidence interval; IV, inverse variance; M-H, Mantel-Haenszel Studies used in the analysis include [10-16].

Subgroup analysis was performed to check whether the administration of corticosteroids in addition to tocilizumab and standard care influenced the results produced, and no significant difference was found in any safety and efficacy outcome among the subgroups except that infection was markedly reduced in those patients who were given corticosteroids (RR=1.29 [0.41, 4.04]; p=0.04; I^2^ =77.5%). The details of other subgroup analyses are given in Table 6 and individual forest plots are given in Figure 6.

**Table 6:** Subgroup analysis by corticosteroid use for safety and efficacy outcomes RR, Relative risk; CI, Confidence Interval; p_subgroups_, p-value for subgroup differences

Outcomes		Corticosteroids given	No corticosteroids given	P_subgroups_	I^2^ (%)
		RR (95% CI)	RR (95% CI)		
Safety outcomes	Mortality	0.70 (0.43, 1.15)	0.19 (0.03, 1.28)	0.2	40.4
	Intensive care unit admission	0.32 (0.02, 5.73)	2.06 (0.41, 10.49)	0.27	18.1
	Post-therapy mechanical ventilation	0.07 (0.00, 1.19)	0.45 (0.14, 1.40)	0.24	27.8
	Post drug infection	2.06 (0.55, 7.66)	0.34 (0.12, 0.95)	0.04	77.5
	Serious adverse events	1.82 (0.99, 3.36)	0.92 (0.40, 2.08)	0.19	42.5
Efficacy outcome	Improved oxygen saturation	1.38 (1.03, 1.86)	1.21 (0.84, 1.75)	0.59	0

## Discussion

Our meta-analysis of seven comparative observational studies, in summary, demonstrated two key findings. Firstly, tocilizumab was superior in lowering mortality rate and need for mechanical ventilation in COVID-19 patients, while insignificant differences in risk of infection and other safety outcomes were seen between the experimental and control group. Secondly, there was a significant improvement in the efficacy outcome, i.e., improved oxygen saturation, in the group treated with tocilizumab. 

Evidence from multiple studies suggests a strong role of Cytokine Release Syndrome (CRS) in the development of ARDS and lung failure [23-26]. A meta-analysis by Coomes et al. revealed a 2.9 times higher mean IL-6 concentration in complicated COVID-19 cases versus non-complicated ones [27]. Since the key cytokine in this hyper-inflammatory response is IL-6, it seems imperative to test the efficacy of IL-6 blockers such as tocilizumab in treating critical COVID-19 patients [24-26]. Tocilizumab is approved by the FDA to treat rheumatoid arthritis, giant cell arteritis, poly-articular juvenile idiopathic arthritis, systemic juvenile idiopathic arthritis, and now CAR-T cell-induced CRS [23-25]. It has proven efficacy in attenuating inflammation and has a well-known long-term safety profile, which makes it suitable for use as an adjunctive immune modulator along with other therapies [23-26].

The baseline characteristics and outcomes of six single-arm, observational studies investigating the safety of tocilizumab therapy in COVID-19 patients are shown in Table 7 [17-22]. All studies show a significantly lower all-cause mortality than that seen in other critically ill COVID-19 cohorts. Almost all the studies show a rapid decline in fever and laboratory markers of inflammation, while there is a statistically significant improvement in subsequent CT scan imaging and oxygen requirements [4,5,17]. This evidence strongly suggests the efficacy of tocilizumab not as a standalone COVID-19 therapy but as an effective adjunct treatment nonetheless, in the subgroup of COVID-19 patients who develop CRS. However, the reliability of these results is limited by their retrospective nature, lack of a control arm for comparison of results, and lack of a defined treatment protocol. That increases the need for other better-designed studies, preferably RCTs.

**Table 7:** Baseline characteristics and outcomes of single-arm studies CVD, Cardiovascular disease; DM, Diabetes mellitus; CKD, Chronic kidney disease; IQR, interquartile range, NR, not reported

Author	Region	Study type	Follow-up period (days)	Total patients	Age in years, median (IQR)	Males, n (%)	CVD, n (%)	DM, n (%)	CKD, n (%)	Chest radiological findings n (%)		Need for invasive ventilation n (%)	Outcomes n (%)	
										Abnormal	Normal		Survival (%)	Need for oxygen support (%)
Alattar et al. [17]	Qatar	Retrospective cohort	14	25	58 (50‐63)	23 (92)	3 (12)	12 (48)	4 (16)	25 (100)	0	21 (84)	22 (88)	14 (60)
Price et al. [18]	Connecticut, USA	Retrospective, single-center	> 21	239	64 (22-99)	125(53)	71 (30)	91 (38)	NR	165 (70)	70 (30)	153 (64)	86%	75 (31)
Morena et al. [19]	Italy, Europe	Retrospective, single-center	34	51	40 (78.4)	60 (50-70)	25 (49.0)	6 (11.8)	NR	50 (98)	1 (2.0)	6 (11.8)	37 (73)	20.4 (40)
Uysal et al. [20]	Istanbul, Turkey.	Retrospective single center	23	12	68 (47-79)	6 (50)	4 (26)	7 (58)	1 (8)	NR	NR	NR	12 (100)	(17)
Luo et al. [21]	Wuhan, China	Retrospective, single-center	7	15	73 (62-80)	12 (30)	NR	NR	NR	NR	NR	NR	12 (80)	NR
Xu et al. [22]	Anhui, China	Retrospective, cohort study	9	21	56.8 (25-88)	18 (85.7)	NR	5/21 (23.8)	1/21 (4.8)	21 (100)	0	2/20 (10.0)	21 (100)	1 (5)

The meta-analysis suggests a significantly lower risk of mortality among the patients who received tocilizumab therapy along with standard therapy compared to those receiving standard therapy alone. This finding is consistent with the findings of most comparative observational studies, including those by Klopfenstein et al. and Capra et al. [10-11]. In contrast to this, Rojas-Marte et al. reported no significant difference in mortality between the two groups; however, the difference was significant once already intubated patients were excluded [12]. Similarly, in the study by Quartico et al., among the subgroup of patients on a ventilator in the tocilizumab-treated group, approximately 50% did not respond to treatment [13]. Furthermore, the meta-analysis showed no significant difference in ICU admissions. These findings strongly suggest that tocilizumab may perhaps be more effective in controlling the early stages of the inflammation and cannot reverse the hyper-inflammatory state once reached. Conversely, the COVACTA trial, which is the first global, randomized, double-blind, placebo-controlled phase III study evaluating the safety and efficacy of tocilizumab in the patients with severe COVID-19 pneumonia, shows no difference in patient mortality at week four [6].

Consistent with the results of the single-arm observational studies, this meta-analysis also demonstrated that a significantly lower number of patients requiring mechanical ventilation following tocilizumab therapy and a significant improvement in their blood oxygen saturation [4-5,26]. This is an important finding in the context of the low availability and high costs of ventilators. Tocilizumab therapy seems to be beneficial in relieving the tremendous economic burden on public health systems.

Tocilizumab is known to pose an increased risk of secondary bacterial/fungal infections due to its immunosuppressive mode of action. However, the pooling of the results demonstrated no significant difference in infections between the two groups. One plausible explanation may be the lower rate of intubation and mechanical ventilation in these patients, eliminating risk from infection-causing procedures. Interestingly, scientists in favor of tocilizumab suggest that the desquamation of alveolar cells, hyaline membrane formation, and pulmonary edema following the hyper-inflammatory state make the lungs more vulnerable to secondary infections, and hence tocilizumab has a protective action [24]. Also, it is noteworthy that tocilizumab is known to cause infections only on continuous dosing, whereas the maximum number of doses administered to COVID-19 patients in these trials is two [26]. Besides, this analysis shows no significant difference in the advent of serious adverse events (SAE) among the two groups. This indicates a low risk-benefit ratio favoring the use of tocilizumab in critically ill COVID-19 patients [23].

Patients were administered corticosteroid therapy as part of the standard regimen in five of the seven cohorts. Corticosteroids have a potent anti-inflammatory effect [28]. The possible overlap of its effects with those of tocilizumab generates significant confounding. The results of the subgroup analysis reported no significant difference in safety and efficacy outcomes except significantly reduced infections in the corticosteroid subgroup (Table 4). Corticosteroids are known to cause immunosuppression and possibly increase the risk of infection when administered in very high doses (>100 mg/day) over an extended period [29]. However, the studies included in this analysis that employ the use of corticosteroid therapy had administered moderate doses of corticosteroids for no more than 10 days; therefore, they pose no increased risk of infections. On the contrary, they may reduce the incidence of post-infectious inflammation.

Limitations

There are several limitations that this meta-analysis attempts to overcome, including the limited sample sizes, non-homogenous sample population, and short duration of studies. To minimize inter-study heterogeneity, the baseline characteristics of both cohorts were adjusted. However, the lack of a uniform standard protocol for treatment and no clear guidelines on the dosing and administration of tocilizumab generate a confounding bias. Moreover, the populations differed in disease severity and the relative time of treatment during the course of the disease.

Tocilizumab appears to improve mortality and oxygen saturation as compared to controls upon pooling of the observational studies; however, the COVACTA trial showed no mortality benefit [6]. The observational studies were of low quality and mostly unadjusted; thus, the mortality benefit seen in these studies may be spurious and due to confounding. On the contrary, the COVACTA trial had a small sample size and low power [6]. Thus, current findings should be viewed cautiously, as neither the RCT nor the meta-analysis of observational studies shows much improvement in the safety outcome, so it is questionable as to where the mortality benefit came from.

## Conclusions

This meta-analysis shows that therapy with tocilizumab lowers the mortality rate and risk of artificial ventilation in COVID-19 patients and improves their oxygen saturation levels. While it has no role in reducing ICU admissions, it does not pose a serious risk of infections and other adverse events. Furthermore, concomitant corticosteroid therapy seems to lower the risk of infections. Similarly, the six single-arm studies show a lower mortality rate with tocilizumab therapy. Tocilizumab is a promising agent to attenuate inflammation and cytokine release, which is the underlying pathophysiology of several COVID-19 cases. However, the poor design and various limitations of the studies render them ineffective in gauging the full extent of its safety and efficacy and thus warrant further research into the use of tocilizumab.
